# Is International Travel an Emerging Issue on Transmission of Beijing Lineage *Mycobacterium tuberculosis*?

**DOI:** 10.1155/2020/9357426

**Published:** 2020-08-28

**Authors:** Pavithra S. Madamarandawala, Srinath Satyanarayana, Collins Timire, Aashifa Yaqoob, Dushantha Madegedara, Dhammika N. Magana-Arachchi

**Affiliations:** ^1^National Institute of Fundamental Studies, Kandy, Hantana Road, Sri Lanka; ^2^International Union Against Tuberculosis and Lung Disease (The Union), Paris, France; ^3^The Union, South-East Asia Office, New Delhi, India; ^4^Ministry of Health and Child Care-National Tuberculosis Control Programme, Harare, Zimbabwe; ^5^Common Management Unit (AIDS, TB & Malaria), Ministry of National Health Services, Regulations and Coordination, Islamabad, Pakistan; ^6^Respiratory Disease Treatment Unit, General Teaching Hospital, Kandy, Sri Lanka

## Abstract

*Mycobacterium tuberculosis* belonging to Beijing sublineage (BL) is associated with high tuberculosis (TB) transmission, multidrug resistance, and adverse treatment outcomes. Sri Lanka experiences an increase in the number of travellers/workers to and from high TB-burden countries, and there is risk of getting BL strains imported into the country. In this context, a cohort study was conducted to assess the prevalence of BL strains among pulmonary tuberculosis (PTB) patients in the Kandy district of Sri Lanka (a popular tourist destination) and its association with patients' sociodemographic and clinical characteristics. The study population included sputum smear-positive PTB patients diagnosed from February 2018–July 2019. Fresh sputum samples were collected for culturing and conducted polymerase chain reaction using BL-specific primers. Among the 101 patients recruited, presence of BL strains could be ascertained in 94 patients of which 24 (26%; 95% CI: 18%–35%) had BL strains. Prevalence of BL strains was higher among those with high sputum smear grades (2+ and 3+) (*P* < 0.05) and those who had travelled abroad (*P* < 0.05). The prevalence was also higher among young people (aged <35 years). Treatment success rates were similar in patients with (83%) and without BL strains (83% vs. 81%; *P* value = 0.8375). The prevalence of BL strains in Kandy, Sri Lanka, was high compared to previously reported figures in Sri Lanka, and the percentage drives closer to the countries in South East Asia. International travel raises itself as an emerging issue in BL transmission urging the need of policies and practices in immigration/emigration strategies. The study findings have the potential to alter the TB epidemiology in the country and might represent the situation in other underexplored countries as well. Therefore, it is important to monitor the trends and factors related to the prevalence of Beijing strains globally and make decisions as a whole.

## 1. Introduction

Seven lineages of *Mycobacterium tuberculosis* (MTB), the bacteria that causes “Tuberculosis” (TB), have been identified till date [1]. Of these, East Asian lineage is inevitably the most widespread, and Beijing sublineage (BL) is its major component [2]. BL was first reported in Beijing, China, in 1995, and shortly, it was spotted/reported in other countries as well [3].

BL gained attention in 1990s due to its links to drug resistance, being “escape variants” of Bacille Calmette–Guerin (BCG) vaccination, higher transmission, and predilection for outbreaks (New York (1990s) and Canaria Island (1993)) [2, 4, 5]. However, studies after 2000s have not shown clear global patterns on these concerns as expected. For instance, while many studies observed the association of BL with multidrug resistant TB (MDR-TB—resistance to at least isoniazid and rifampin) as in Indonesia, Iran, Pakistan, and Georgia, some studies showed no such association [4, 6, 7]. Given this uncertainty, the current global concern is to assess the BL prevalence, study its associations with patient's characteristics, assess the timely changes, and make informed decisions [8].

Sri Lanka, a developing country in South Asia, has a moderate TB burden (estimated incidence rate (2017) = 64/100,000 population) [7]. In 2003, 8.8% of the TB patients tested belonged to BL [9]. However, this study was conducted among a group of Sri Lankans who travelled abroad and may not be a good representative. During 2005–2006, 14.3% BL prevalence was reported from Colombo, Sri Lanka, and in Kandy, Sri Lanka, it was 22.4% in 2012 [10, 11]. After the end of the 30-year civil war in 2009 and attaining politicoeconomic stability, the country experienced an increase in the number of travellers/workers to and from high TB-burden countries for tourism and construction workforce. Sri Lanka's TB incidence rate has been stable for the last decade [12]. However, there are concerns that highly virulent BL strains may get imported from the neighboring countries as was seen in Canaria Islands [5]. Therefore, there is a need to assess the current BL prevalence and monitor its trends in various geographic settings of the country [11]. In this context, we undertook a study in Kandy (a popular tourist destination) with the aim of understanding the prevalence, patient characteristics, and treatment outcomes associated with BL strains among tuberculosis patients in Kandy, Sri Lanka.

## 2. Materials and Methods

### 2.1. Ethics

Approval was obtained from the Ethics Advisory Group, International Union against Tuberculosis and Lung Disease (19/19) and Ethical Review Committee, General Teaching Hospital, Kandy (GTHK), Sri Lanka. Data were collected after obtaining written informed consent from each patient.

### 2.2. Study Design

We conducted a cohort study involving both primary and secondary data collection.

### 2.3. Study Setting

#### 2.3.1. General Setting

**Figure 1:**
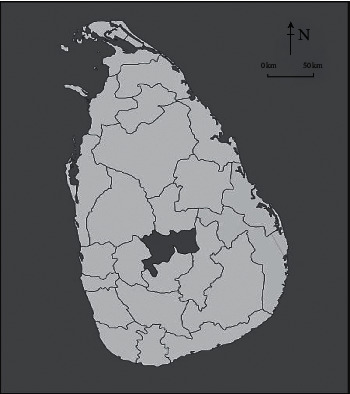
A map of Sri Lanka depicting the study area: Kandy district.

The study was conducted in Kandy, the capital district in the central province, Sri Lanka (Figure 1). It is the second most important district in the country with a total population of ∼1.4 million and a daily transient population of ∼100,000 people to the city [13].

#### 2.3.2. Hospital Setting

GTHK is a tertiary care government hospital which has a separate chest clinic where all the TB patients diagnosed in Kandy district are registered. There are two consultant respiratory physicians (CRPs) who hold clinics on alternate days. They enroll all PTB patients diagnosed both in the hospital and district clinics outside the hospital, under their care. Both physicians receive approximately similar patients in terms of numbers and patient characteristics. Sputum microscopy and, depending on the physician's decision, culture, and Xpert MTB/Rif assay are conducted for TB diagnosis. All sputum smear-positive patients are registered and initiated on a six-month treatment regimen. Suspected MDR-TB patients are transferred to the national hospital for respiratory diseases. There is directly observed treatment, and a treatment booklet is maintained for each patient. Treatment adherence is monitored ensuring that patients attend their scheduled follow-up hospital visits. At the end of treatment, all TB patients are categorized into standard TB treatment outcomes as per the World Health Organization (WHO) guidelines (see Table S1 in Supplementary Materials for treatment outcome definitions for TB patients) [14].

### 2.4. Study Population

The study population included all sputum smear-positive PTB patients diagnosed at GTHK, Sri Lanka, from February 2018 to July 2019 (*n* = 101). Due to operational and administrative reasons, we had permission to recruit patients enrolled into care by one of the two CRPs.

### 2.5. Sputum Sample Collection and Processing

Fresh sputa were collected from each patient and decontaminated using the modified Petroff's method [15]. The resultant pellet was used to inoculate Lowenstein–Jensen (LJ) media, containing thiophene-2-carboxylic acid hydrazide (TCH) and *para*-nitrobenzoic acid (PNB) to differentiate MTB and nontuberculous mycobacteria. The media were incubated at 37^o^C and 28^o^C in light and dark conditions, and growth was observed for 4–8 weeks. Acid-fast staining was performed on the culture isolates, and those which gave positive results were suspected to be MTB.

### 2.6. Molecular Testing

Deoxyribonucleic acid (DNA) was extracted from suspected MTB isolates using the standard CTAB (N-cetyl-N,N,N-trimethylammonium bromide) method and from culture-negative sputa using modified Booms' method [16, 17]. DNA was amplified in a multiplex polymerase chain reaction (PCR) using a real-time instrument (RotorGeneQ, Germany). Primers used were specific for the Rv0627c gene of MTB complex which possesses a single-nucleotide polymorphism, cytosine to guanine at position 426, unique to BL (Box 1) [18]. The standard strain, H37Rv, and a confirmed Beijing strain were used as the non-Beijing and Beijing controls. Amplicons were run on a 2% agarose gel incorporated with ethidium bromide and visualized using a gel documentation system (Syngene, UK). BL strains were identified by the two distinct bands (261 bp and 163 bp) that travel the same distance as the base pairs in the positive control. DNA that did not show either of the two bands in multiplex PCR was PCR-amplified conventionally using primers Pt8 and Pt9 which partially amplifies *IS6110*, an insertion element unique to MTB complex (Box 1) [19].

### 2.7. Data Variables, Sources, and Data Collection

Sociodemographic, socioeconomic, behavioural, and clinical information was collected using an interviewer-administered questionnaire. Data on treatment outcomes, sputum grading, and MTB lineage were collected from treatment booklets and laboratory registers. The key outcome variable was MTB lineage (Beijing/non-Beijing lineage).

### 2.8. Data Analysis

Data were double-entered, validated using EpiData v3.1, and were analyzed using EpiData v2.2.2.183 (EpiData Association, Odense, Denmark). Frequencies were calculated for categorical variables disaggregated by Beijing/non-Beijing status. Continuous variables were summarized using means and standard deviation. Chi-square test was used to assess the differences in the proportions. Prevalence ratio and its 95% confidence intervals (CIs) were calculated to assess the association between BL infection and sociodemographic and clinical factors. Level of significance was set at *P* < 0.05.

## 3. Results

**Figure 2:**
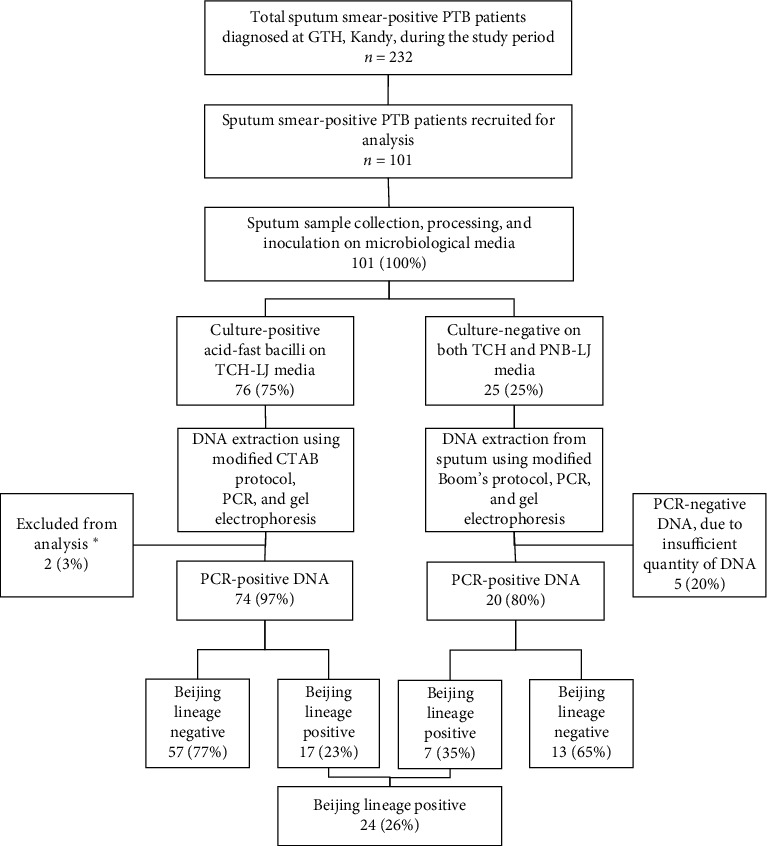
Prevalence of Beijing lineage strains among sputum smear-positive pulmonary tuberculosis (PTB) patients diagnosed in a tertiary care hospital in Kandy, Sri Lanka, during February 2018–July 2019. PCR-negative DNA, due to insufficient quantity of DNA. PTB: pulmonary tuberculosis, DNA: deoxyribonucleic acid, CTAB; cetyl trimethylammonium bromide, PCR: polymerase chain reaction, LJ: Lowenstein–Jensen media, TCH: thiophene-2-carboxylic acid hydrazide, PNB: *para*-nitrobenzoic acid. ^*∗*^Beijing/non-Beijing status could not be ascertained as they did not produce any band in Beijing multiplex PCR. However, they were confirmed as MTB upon conventional PCR.

**Figure 3:**
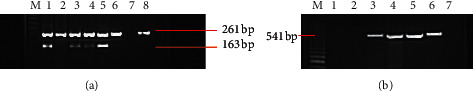
Agarose gel profiles displaying the PCR products of Beijing multiplex PCR and conventional PCR. (a) Agarose gel profile displaying the PCR products of Beijing multiplex PCR (M; 50 bp DNA marker; *lanes* 1, Beijing lineage positive control (a confirmed Beijing strain); 2–6, amplicons from culture isolates; 7, negative control (water); 8, non-Beijing control (the standard *Mycobacterium tuberculosis* strain H37Rv)). (b) Agarose gel profile displaying the PCR products of Beijing multiplex PCR and conventional PCR for the two culture-positive isolates in which the Beijing/non-Beijing status could not be ascertained (M; 50 bp DNA ladder; *lanes* 1, Beijing multiplex PCR product of culture isolate (a); 2, Beijing multiplex PCR product of culture isolate (b); 3 and 4, positive controls (the standard *Mycobacterium tuberculosis* strain H37Rv); 5, conventional PCR product of culture isolate (a); 6, conventional PCR product of culture isolate (b); 7, negative control (water)).

In total, 101 sputum smear-positive PTB patients were recruited, and 76 patients' (75%) sputa yielded acid-fast stain positive cultures on TCH-LJ (Figure 2). Out of 76, Beijing/non-Beijing status could not be ascertained in two isolates as they did not produce amplicon/s in multiplex PCR. However, they were confirmed as MTB upon conventional PCR (Figure 3). Among the culture-negative group (*n* = 25), five were excluded from further analysis due to insufficient DNA quantity. Overall, BL status could be ascertained in 94 samples, out of which 24 (26%; 95% CI: 19%–35%) had BL strains (7 (35%) from culture-negative sputa and 17 (23%) from culture-positive isolates; prevalence ratio in culture-negative : culture-positive samples = 1.52 (95%CI: 0.74–3.16)).

The demographic, socioeconomic, behavioural characteristics, and the BL prevalence among various patient groups are summarized in Table 1. Overall, the mean age (standard deviation) was 44 (16) years, and majority 66% were males. On bivariable analysis, the relative prevalence of BL strains was higher among those who had travelled abroad, young people, females, those with higher educational grades, those whose profession was business, and in those without history of alcohol or smoking. However, except travel history (*P*=0.0117), these associations were not statistically significant (Table 2).

Table 3 summarizes the patients' clinical characteristics disaggregated by the BL status. 67% of patients with BL strains had high sputum grades (Chi-square *P* value = 0.0441). In addition, other clinical characteristics were similar in all patients of the 94 patients, and treatment outcomes were available for 64 patients by the end of September 2019. All were treated with first-line anti-TB drug regimen, and their treatment outcomes are summarized in Table 4. The treatment success rate was similar in all patients.

## 4. Discussion

The study had four important findings. Approximately, 26% of PTB patients in Kandy were infected with BL strains. BL-infected patients have higher sputum smear grade, and a majority have an out-of-country travel history, and among all, the treatment outcomes were similar.

First, the prevalence of BL strains among PTB patients was higher compared to the previous figures in Sri Lanka [9, 10]. This could be due to an increasing trend within Kandy. However, it is not clear whether this represents variations in the BL distribution in our country, as there are no studies reported from regions other than Colombo and Kandy. Nevertheless, results are comparable with Nepal (31%), Indonesia (35%), and Singapore (26%), lower than China (66%), Japan (73%), and Vietnam (47%) but, higher than Afghanistan (10%), Malaysia (17%), and India (3–11%) [20–24].

Second, BL infection was found to be more common among those who have an out-of-country travel history. This suggests that the infection might have occurred while being out-of-country. The reported BL prevalence of the countries visited by those who had BL infection were Singapore (26%), India (3–11%), Saudi Arabia (4.5%), and Malaysia (17%) [20, 21]. Middle-East Asia, being a region that provides job opportunities as labourers, bears nearly 90% of Sri Lankan labour migrants out of 212,162 persons in 2017. Around 9.2% of the total labour migrants were from Kandy district, and it was the third highest district representative [25]. The first documented case of MDR-TB in a foreign-born migrant worker in Sri Lanka also had travelled to Kuwait and India [26]. In addition to workforce, Saudi Arabia is also visited for pilgrimage as was seen in our study. Apart from Middle-East, Asian countries including Japan, Korea, and Malaysia have become the new labour migrant destination countries for people from developing countries. Moreover, labour migrants from South East Asia are prominently seen in construction projects in developing countries like Sri Lanka [25]. In this context, the inflow of virulent MTB strains through migration should not be underestimated. However, significant correlation of duration of their stay abroad and purpose of travel was not observed, and further testing is required to confirm the strain origin.

Third, a significantly larger proportion of patients with BL strains had high sputum smear grades. Sputum grading is a direct indicator of bacillary load, and higher grades are associated with higher bacillary load, thereby increasing risk of TB transmission [27]. Some studies have reported higher bacillary load among BL strains, whereas others have reported similar bacillary loads [28, 29]. This perhaps explains the reasons for higher TB transmission linked to BL.

Fourth, the BL infection was more common among young people. This is consistent with studies done in Vietnam, Hong Kong, and Bangladesh [30, 31]. TB among youth represents recent transmission when compared to adults in whom reactivation of previous infection is more common [4]. Thus, we hypothesize that non-Beijing strains were more common earlier, and it may be getting replaced with Beijing strains off late. Whether our hypothesis is true or not needs to be assessed in future studies.

Lastly, the clinical features and the TB treatment outcomes were similar in all patients. This is in contrast to the previous knowledge from a meta-analysis which had concluded that BL strains are associated with unfavourable treatment outcomes [29, 32].

The study had a few limitations. For two MTB isolates, we could not ascertain Beijing/non-Beijing status, which could perhaps be due to deletion/mutation in the Rv0627c gene. The other limitation was the small sample size because of which the 95% CIs for assessing the differences in the patient characteristics with and without BL strains are wide and imprecise. Therefore, we cannot conclude with statistical certainty whether there were or there were no differences in the patient characteristics. Given this scenario, we strongly feel that it is important to take note of the magnitude and direction of central values of the association measures, which we believe would not have changed if the study has been conducted with larger sample size. We recruited a subsample of patients who nearly constituted half of the PTB patients diagnosed in Kandy during the study period. Therefore, the findings are generalizable to all PTB patients in Kandy. Moreover, we ensured data quality by prospective data collection which adds much strength to our findings.

## 5. Conclusions

In conclusion, the BL prevalence in Kandy, Sri Lanka, was high accounting 26% of PTB patients. The value is much closer to the percentages reported from South East Asia, the hub of BL MTB strains. BL-infected patients appear to be young, likely to contain higher sputum bacillary load, and a majority have an out-of-country travel history. We assume that we would expect similar observations even in other underexplored countries, and these features have the potential to alter the TB epidemiology as a whole. Findings further emphasize the importance of policies and practices to be followed in terms of immigration/emigration not only in Sri Lanka but also globally. Hence, it is important to monitor the trends in the prevalence of Beijing strain and associated risk factors globally and take necessary actions to combat the issue.

## Figures and Tables

**Algorithm 1:**
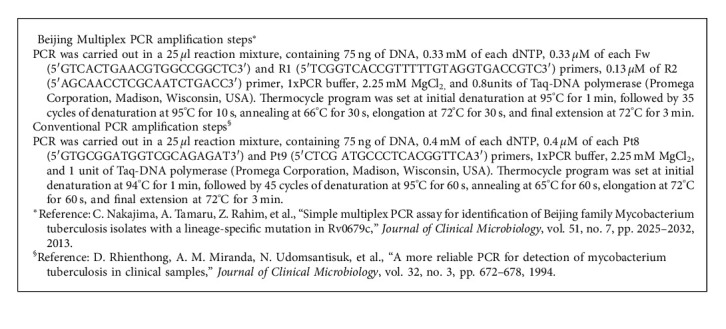
PCR amplification steps.

**Table 1:** Demographical, socioeconomic, and behavioural factors associated with Beijing lineage infection among TB patients diagnosed in a tertiary care hospital in Kandy, Sri Lanka, during February 2018–July 2019.

Variable	Total	TB patients with Beijing lineage strains		PR (95% C.I.)
	*N*	*n*	(%)^*∗*^	
	94	24	(26)	
*Age groups* (*years*)				
15–34	27	10	(37)	Reference
35–64	58	13	(22)	0.61 (0.30–1.20)
≥65	9	1	(11)	0.30 (0.04–2.03)

*Gender*				
Male	62	15	(24)	0.86 (0.42–1.75)
Female	32	9	(28)	Reference

*Type of residency*				
Own house	92	23	(25)	Reference
Not own house	2	1	(50)	2.00 (0.48–8.36)

*Out-of-country travel history*				
Not travelled	81	17	(21)	Reference
Travelled	13	7	(54)	2.57 (1.33–4.95)

*Level of education*				
Up to primary	13	2	(15)	Reference
Secondary	59	13	(22)	1.43 (0.37–5.59)
High-school/university	22	9	(41)	2.66 (0.68–10.47)

*Occupation*				
Professional	20	4	(20)	0.74 (0.25–2.19)
Labourer	34	7	(21)	0.76 (0.31–1.91)
Business	14	6	(43)	1.59 (0.66–3.82)
Not employed	26	7	(27)	Reference

*Monthly income (LKR)*				
No income	27	8	(30)	1.85 (0.64–5.40)
≤10,000	25	4	(16)	Reference
>10000	42	12	(29)	1.79 (0.65–4.94)

*Use of alcohol*				
Yes	45	11	(24)	0.92 (0.46–1.84)
No	49	13	(27)	Reference

*Smoking*				
Yes	38	10	(26)	1.05 (0.52–2.12)
No	56	14	(38)	Reference

^*∗*^Row percentages. PR = prevalence ratio, C.I. = confidence interval, LKR = Sri Lankan rupees. *Level of education:* up to primary: grades 1–5, secondary: grades 6–11 (up to ordinary level examination), and high-school: grades 12-13 (up to advanced level examination). *Occupation:* labourer includes industry and estate labourers. Professional includes skilled workers in the following categories: government officers, hospital staff, tourist guidance, hotel management etc.

**Table 2:** Out-of-country travel information of TB patients diagnosed in a tertiary care hospital in Kandy, Sri Lanka, between February 2018 and March 2019 disaggregated by their Beijing lineage strain status.

Out-of-country travel history	TB patients infected with Beijing lineage (*n*)	TB patients infected with non-Beijing lineage (*n*)	*P* value
Travelled	7	6	0.0117
Travelled to one country only	6	4	
Travelled to two countries	1	2	

*Country travelled*			
Dubai	0	1	
Germany	0	1	
Jordan	0	1	
Kuwait	3	0	
Malaysia	1	1	
Saudi Arabia	2	2	
Singapore	1	0	
India	1	0	
Japan	0	1	
Maldives	0	1	

*Duration of visit abroad*			
Less than one year	2	1	
1–5 years	3	2	
6–10 years	0	0	
10–15 years	1	1	
16–20 years	1	0	
21–25 years	0	1	
Not recorded	0	1	

*Purpose of travel*			
Labour migrant	5	4	
Pilgrimage	2	1	
Tourism	1	0	
Other	0	1	

*P* value < 0.05.

**Table 3:** Clinical presentation among pulmonary tuberculosis patients in a tertiary care hospital in Kandy, Sri Lanka (February 2018–July 2019).

	TB patients infected with Beijing lineage		TB patients infected with non-Beijing lineage		Total
	*n*	(%) §	*n*	(%)§	
Total	24		70		
*Sputum grade*					
Low grade (scanty/1+)	8	(33)	40	(57)	48
High grade (2+/3+)	16	(67)	30	(43)	46

*Symptoms*					
Cough	23	(25)	69	(75)	92
Fever	14	(58)	33	(47)	47
Headache	0	(0)	11	(17)	11
Haemoptysis	4	(17)	16	(23)	20
Loss of weight	13	(54)	38	(54)	51
Body pain	2	(11)	3	(6)	5

*Type of TB patient*					
New	24	(100)	67	(96)	91
Relapse	0	(0)	3	(4)	3

*Presence of comorbidities*					
Diabetes mellitus	7	(29)	16	(23)	23
Asthma	0	(0)	4	(6)	4

TB: tuberculosis. *P* value < 0.05. § = column percentages. *Type of TB patient:* new: never been treated for TB or have taken anti-TB drugs for less than 1 month; relapse: previously treated for TB were declared cured or treatment completed at the end of their most recent course of treatment and are now diagnosed with a recurrent episode of TB (either a true relapse or a new episode of TB caused by reinfection) (Reference: WHO, *Definitions and Reporting Framework for Tuberculosis-2013 Revision*, World Health Organization, Geneva, Switzerland, 2013.).

**Table 4:** Comparison of treatment outcome of 64 TB patients diagnosed in a tertiary care hospital in Kandy, Sri Lanka, between February 2018 and March 2019 disaggregated by their Beijing lineage strain status.

Treatment outcome^*∗*^	TB patients infected with Beijing lineage		TB patients infected with non-Beijing lineage		*P* value
	*n*	(%)§	*n*	(%)§	
Total	12		52		
Favorable					
Cured	10	(83)	42	(81)	0.8375
Unfavorable	2	(17)	10	(19)	
Died	0	(0)	3	(6)	
Not evaluated	2	(17)	7	(13)	

TB: tuberculosis. § = column percentage. *P* value < 0.05. *Note.* The table gives data only of the patients whose treatment outcome information were available by September 2019 (*n* = 64). ^*∗*^Reference: WHO, *Definitions and Reporting Framework for Tuberculosis-2013 Revision*, World Health Organization, Geneva, Switzerland, 2013.

## Data Availability

Data generated during the study will be available from the corresponding author upon reasonable request.

## Supplementary Materials

Supplementary Materials Table S1: treatment outcome definitions for TB patients (excluding patients treated for RR-TB or MDR-TB).
