# Supplementary material for: Is International Travel an Emerging Issue on Transmission of Beijing Lineage Mycobacterium tuberculosis?
Source: J Trop Med. 2020 Aug 28;2020:9357426. doi: 10.1155/2020/9357426 (PMC7474789; doi:10.1155/2020/9357426)
Supplement: Supplementary Materials — Table S1: treatment outcome definitions for TB patients (excluding patients treated for RR-TB or MDR-TB). [file 9357426.f1.docx]

**Supplementary files:**

**Table S1: Treatment outcome definitions for TB patients (excluding patients treated for RR-TB or MDR-TB).**

| **Outcome** | **Definition** |
| --- | --- |
| Cured | A pulmonary TB patient with bacteriologically confirmed TB at the beginning of treatment who was smear- or culture-negative in the last month of treatment and on at least one previous occasion |
| Died | A TB patient who dies for any reason before starting or during the course of treatment |
| Not evaluated | A TB patient for whom no treatment outcome is assigned. This includes cases “transferred out” to another treatment unit as well as cases for whom the treatment outcome is unknown to the reporting unit. |

*TB: tuberculosis, RR-TB: rifampin resistant tuberculosis, MDR-TB: multi drug resistant tuberculosis*

*(Reference: WHO. Definitions and reporting framework for tuberculosis - 2013 revision. World Health Organization, Geneva, Switzerland. 2013. 3–7 p.)*
